# An MRI-Derived Formula for Estimating the Native Joint Line Position in the Presence of Distal Femoral Bone Loss

**DOI:** 10.7759/cureus.73707

**Published:** 2024-11-14

**Authors:** Reuben P Rao, Angel Thien Thien Lim, Jade Pei Yuik Ho, Lik Han Ong, Faris Kamaruddin

**Affiliations:** 1 Department of Orthopaedics, Sarawak General Hospital, Kuching, MYS; 2 Department of Radiology, Sarawak General Hospital, Kuching, MYS; 3 Department of Orthopaedics, Kuala Lumpur Hospital, Kuala Lumpur, MYS; 4 Department of Orthopaedics, Faculty of Medicine and Health Sciences, Universiti Malaysia Sarawak, Kuching, MYS

**Keywords:** adductor tubercle, bone loss in tka, joint line restoration, revision total knee arthroplasty, transepicondylar width

## Abstract

Background

In the presence of distal femoral condyle bone loss, estimation and restoration of the joint line (JL) position can be guided by extraarticular bony landmarks with the aid of mathematical formulas that rely on the innate correlations between periarticular measurements. To prevent JL elevation, the formula should incorporate the thickness of distal femoral articular cartilage. The aim of this study was to derive a formula to estimate native JL position.

Methods

One hundred and fifty knee magnetic resonance imaging (MRI) studies belonging to 150 patients were chosen from a database of scans. Multiple periarticular measurements were taken. Based on the strongest correlation between measurements, linear regression analysis was used to derive a regression equation to estimate the JL position. This formula was then tested to determine its accuracy and reliability in estimating the JL.

Results

Using the Pearson correlation test, the strongest correlation was identified to be between adductor tubercle to joint line distance (ATJL) and transepicondylar width (TEW) with r = 0.723, p <.001. Using linear regression analysis, the following regression equation was obtained: ATJL in millimetres = 0.53 (TEW in millimetres) + 2.4mm. This formula estimated the JL within 4 mm of the native JL in 86% of measured knees and within 8 mm in 100% of measured knees. The mean difference between calculated ATJL and measured ATJL was 2.43 mm with a standard deviation of 1.94 mm.

Conclusion

The current formula (ATJL = 0.53(TEW) + 2.4mm) reliably estimates native JL distance from the adductor tubercle (AT) to within a clinically significant range, using femoral TEW.

## Introduction

A technical goal and principle of all total knee arthroplasty (TKA) is the restoration of the tibio-femoral joint line (JL) [1]. Restoration of the JL affects the kinematics, function, and symptoms of the prosthetic knee. In most primary TKAs, JL restoration is achieved by performing a measured resection of the distal femur. This relies on the preservation of distal femoral condylar bone to guide bony resection and replacement. In revision knee arthroplasty, however, distal femoral condylar bone loss is common and JL restoration using conventional methods may not be possible. In such cases, estimation and restoration of the JL can be guided by the extraarticular bony landmarks. Bony landmarks can be used to estimate native JL position either with the help of measurements taken from imaging of the contralateral knee, by using a fixed distance from the landmark, which is usually based on a population mean for a given measurement [2], or by applying a mathematical formula that estimates the JL position as a function of an intraoperatively measurable parameter.

The accuracy of measurements taken from imaging of the contralateral knee is highly dependent on magnification, while the accuracy of fixed distances is affected by variations in knee size. Therefore, over the past decade and a half, formulas and ratios for the estimation of JL have risen in popularity. These formulas are derived from observed correlations between 2 or more knee measurements. One such formula estimates adductor tubercle to joint line distance (ATJL) as a function of femoral transepicondylar width (TEW) and was first described based on measurements using plain radiographs [3, 4].

The key limitation to the use of plain radiographs is the inability to account for distal femur condylar articular cartilage thickness and therefore a predisposition to underestimation of ATJL and JL elevation. This shortcoming can be obviated by deriving a formula based on magnetic resonance imaging (MRI), cadaveric, or intraoperative measurements. To date, though, there is a limited number of such studies, which include a relatively modest number of knees. To the best of our knowledge, there has only been one other study that has defined the correlation between ATJL and TEW using knee MRIs [5].

These limitations point to the need for a larger study to obtain a formula for the accurate estimation of native JL position that accounts for articular cartilage thickness of the distal femoral condyles. The aim of this study was to derive such a formula from measurements taken on knee MRIs.

## Materials and methods

This study consists of two parts. The first involves the measurement of knee MRIs and identification of the intraoperatively reproducible measurement with the strongest linear correlation to joint line position. The primary outcome measure was the strength of this correlation. The second part of the study is the derivation of a mathematical formula that accurately and reliably estimates joint line position based on the previously identified correlation. The primary outcome measure of the second part is the accuracy of the formula.

One hundred and fifty knee MRI studies belonging to 75 males and 75 females were chosen from a database of MRI scans performed prior to this study for various reasons. All MRI scans were performed using a GE Signa LX 1.5 Tesla MRI scanner (GE Healthcare, Chicago, IL, USA). To ensure that our analysis was representative of normal, skeletally mature anatomy, knees with evidence of prior surgery, tumours, chondral injury, osteoarthritis, prior fractures, or skeletal immaturity were excluded. Measurements were made on digital T1-weighted MRI images using commercial software designed for radiographic measurements, OsiriX DICOM viewer (Pixmeo SARL, Bernex, Switzerland).

**Figure 1:**
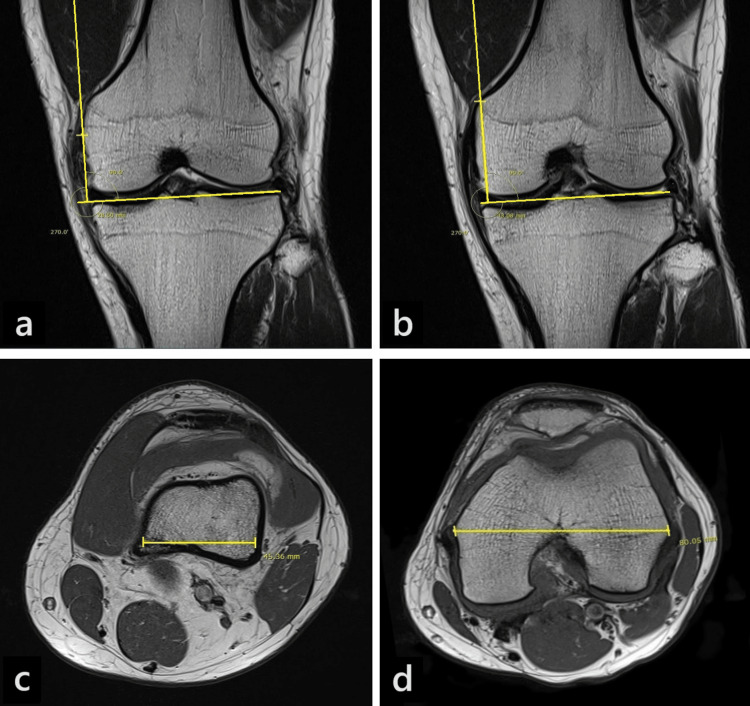
Measurements on the MRI scans a) Measurement of MEJL on coronal slice 1; b) Measurement of ATJL on coronal slice 2; c) Measurement of ATW on axial slice 1; d) Measurement of TEW on axial slice 2. MEJL: medial epicondyle to joint line distance, ATJL: adductor tubercle to joint line distance, ATW: width of the femur at the level of the adductor tubercle, TEW: transepicondylar width

From each T1-weighted MRI study, two coronal slices and two axial slices were selected (Figure 1). The tibio-femoral JL was drawn as a tangent connecting the most distal points of the medial and lateral femoral condyles articular cartilage. The JL was then superimposed onto each of the selected coronal slices.

Coronal slice one

The coronal slice at the most prominent point of the medial epicondyle was selected. Medial epicondyle to joint line distance (MEJL) was measured as the perpendicular distance between the most prominent point of the medial epicondyle and the superimposed JL (Figure 1a).

Coronal slice two

The coronal slice containing the tip of the adductor tubercle (AT) was selected. The tip of the AT was identified as the most distal point on the medial supracondylar slope of the femur before the medial drop-off. The ATJL was measured as the perpendicular distance between the tip of the AT and the superimposed JL (Figure 1b).

Axial slice one

The axial slice containing the tip of the AT was selected. On this slice, the width of the femur at the level of the adductor tubercle (ATW) was measured as the bicortical width of the femur that passes through the AT in the coronal plane (Figure 1c).

Axial slice two

The axial slice containing the most prominent point of the ME was selected. The TEW was measured as the distance between the most prominent points of the medial and lateral epicondyles on this slice (Figure 1d).

Statistical analysis

All demographic and MRI measurement data were recorded using Microsoft® Excel® for Microsoft 365 MSO, Microsoft Corporation, Redmond, WA, USA.

All initial measurements were made by a single author (A.L.). To determine the intra- and interobserver reliability of the measurements, one month after the initial measurements were made, a randomly selected sample of 30 knee MRIs was evaluated by two authors (A.L. and R.R.) independently and blinded to the initial measurements. From this sample, ATJL, MEJL, and TEW measurements were made. Intraclass and interclass correlation coefficients for ATJL, MEJL, and TEW were calculated using two-way absolute agreement (Tables 1, 2). The intraclass correlation coefficients for ATJL, MEJL, and TEW were 0.974, 0.964, and 0.973, respectively. The interclass correlation coefficients for ATJL, MEJL, and TEW were 0.945, 0.963, and 0.979, respectively. All the inter- and intraobserver reliabilities were considered “excellent”.

**Table 1:** Interclass correlation ATJL: adductor tubercle to joint line distance; MEJL: medial epicondyle to joint line distance; TEW: transepicondylar width

Variable	Cronbach’s alpha	Interclass correlation coefficient	95% Confidence interval	
			Lower limit	Upper limit
ATJL	0.944	0.945	0.885	0.974
MEJL	0.962	0.963	0.923	0.982
TEW	0.978	0.979	0.955	0.990

**Table 2:** Intraclass correlation ATJL: adductor tubercle to joint line distance; MEJL: medial epicondyle to joint line distance; TEW: transepicondylar width

Variable	Cronbach’s alpha	Intraclass correlation coefficient	95% Confidence interval	
			Lower limit	Upper limit
ATJL	0.973	0.974	0.946	0.988
MEJL	0.963	0.964	0.924	0.983
TEW	0.972	0.973	0.943	0.987

Due to the normal distribution of all measurements, the Pearson correlation test was chosen to calculate the correlation coefficient for each of the following four pairs of measurements: ATJL and TEW, ATJL and ATW, MEJL and TEW, and MEJL and ATW

The pair with the strongest linear correlation was further analysed using linear regression analysis to define the relationship between the two measurements in the form of a mathematical formula. The derived formula was then applied to all previously measured knee MRIs to test for accuracy and reliability by comparing the formula calculated for ATJL (cATJL) to ATJL as measured from the MRI (mATJL). Results were recorded as absolute values to represent joint line alteration rather than elevation or depression.

Joint line estimation was also performed using the fixed distance method, whereby joint line position was estimated using a previously reported fixed distance from the medial epicondyle to the medial JL. This joint line estimation was compared to MEJL as measured on MRI (mMEJL). The accuracy and reliability of the formula were then compared to those of the fixed distance method.

Statistical analysis was performed using IBM SPSS Statistics for Windows, Version 24.0 (IBM Corp., Armonk, NY, USA).

Ethical considerations

Ethical approval was obtained from the Medical Research & Ethics Committee, Ministry of Health, Malaysia, prior to the start of this study (NMRR ID: 23-03435-BGN (IIR)). Each patient’s identifying details were omitted from the collected data and replaced by a unique serial number assigned for the purpose of the study. All records and data were kept confidential, and all precautions were taken to maintain data confidentiality. This study was not supported by external funding.

## Results

The combined mean age of participants was 31.8 years (range 16-53 years). For female subjects, the mean age was 31.5 years (range 16-53 years) and for male subjects, it was 32.1 years (range 16-50 years) with no significant difference of age between sexes. Knees were divided almost equally, with 76 and 74, left and right knees measured respectively. There were significantly more right knees measured for females and more left knees measured for males; however, we do not expect this imbalance to skew the results of this study (Table 3).

**Table 3:** Demographic data and MRI measurements of subjects TEW: transepicondylar width; ATW: width of the femur at the level of the adductor tubercle; MEJL: medial epicondyle to joint line distance; ATJL: adductor tubercle to joint line distance

	Series (n=150)	Female (n=75)	Male (n=75)
Age mean and range (years)	31.8 years (16-53)	31.5 years (16-53)	32.1 years (16-50)
Left/right (n)	76/74	29/46	47/28
TEW mean and range (mm)	74.8mm (61-89)	70.2mm (61-79)	79.5mm (71-89)
ATW mean and range (mm)	63.9mm (53-77)	60.8mm (53-70)	67.1mm (57-77)
MEJL mean and range (mm)	24.6mm (18-39)	23.2mm (18-37)	26.1mm (18-39)
ATJL mean and range (mm)	42.2mm (27-52)	39.5mm (27-46)	44.8mm (34-52)

The mean MEJL was measured to be 23.2 mm (range 18-37 mm) for females and 26.1 mm (range 18-39 mm) for males, which was significantly different with p <.001. The mean ATJL was 39.5 mm (range 27-46 mm) for females and 44.8 mm (34-52 mm) for males, which was also significantly different, with p <.001. The mean combined MEJL and ATJL were 24.6 mm and 42.2 mm, respectively. The combined mean TEW was 74.8 mm (range 61-89 mm) and the mean ATW was 63.9 mm (range 53-77 mm). Both TEW and ATW also showed significant differences between sexes with p<.001.

**Figure 2:**
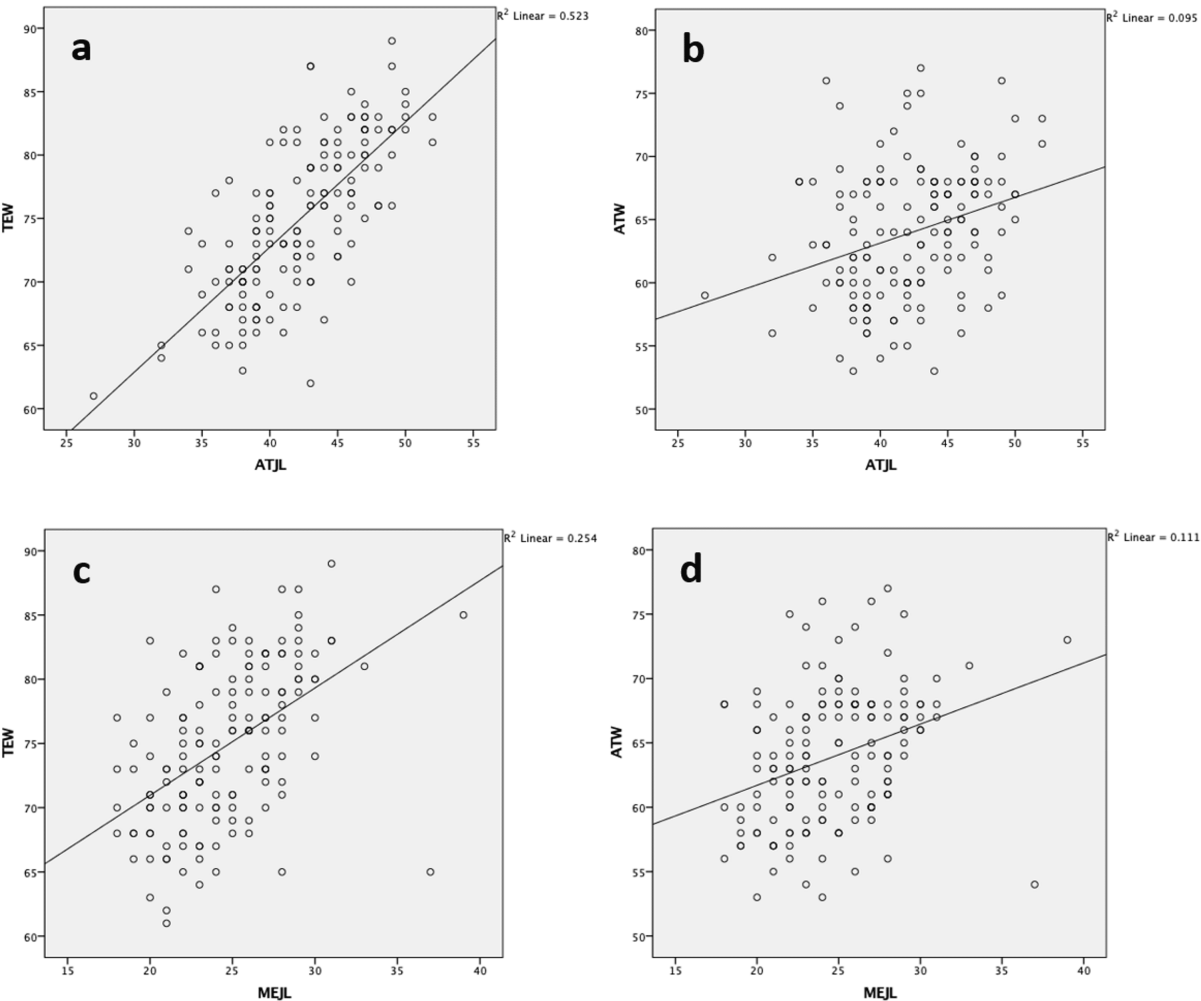
Linear regression plots between TEW and ATJL (a), between ATW and ATJL (b), between TEW and MEJL (c), and between ATW and MEJL (d) ATW: width of the femur at the level of the adductor tubercle; ATJL: adductor tubercle to joint line distance; MEJL: medial epicondyle to joint line distance; TEW: transepicondylar width

Using Pearson correlation, a significant correlation was found between ATJL and both ATW and TEW. Similarly, a significant correlation was found between MEJL and both ATW and TEW. However, only the ATJL and TEW pairs showed a strong correlation, with r = 0.723. All other pairs had weak correlations, with r values between 0.308 and 0.504 (Table 4; Figure 2).

**Table 4:** Results of the Pearson correlation test for four pairs of measurements ATW: width of the femur at the level of the adductor tubercle; ATJL: adductor tubercle to joint line distance; MEJL: medial epicondyle to joint line distance; TEW: transepicondylar width

		ATJL	MEJL
ATW	Pearson correlation	0.308	0.333
	Sig. (two-tailed)	<0.001	<0.001
	N	150	150
ATJL	Pearson correlation	1	0.463
	Sig. (two-tailed)		<0.001
	N	150	150
MEJL	Pearson correlation	0.463	1
	Sig. (two-tailed)	<0.001	
	N	150	150
TEW	Pearson correlation	0.723	0.504
	Sig. (two-tailed)	<0.001	<0.001
	N	150	150

Using linear regression analysis, the following regression equation was obtained:

$ATJL = 0.53(\text{TEW}) + 2.4 \, \text{mm}$

where ATJL in millimetres is the dependent variable, TEW in millimetres is the independent variable, and 2.4mm is the Y-axis intercept.

The mean difference between the calculated ATJL and measured ATJL was 2.43 mm with a standard deviation of 1.94 mm. This formula was able to estimate joint line to within 4 mm of the native JL position in 129 (86.0%) of measured knees and within 8 mm in 150 (100%) of measured knees (Table 5).

**Table 5:** Difference between formula calculated ATJL and measured ATJL cATJL: adductor tubercle to joint line distance as calculated using formula; mATJL: adductor tubercle to joint line distance as measured on magnetic resonance imaging; ATJL: adductor tubercle to joint line distance

Absolute difference between cATJL and mATJL	Series (n=150)	Female (n=75)	Male (n=75)
Mean (mm)	2.43mm	2.32mm	2.53mm
SD (mm)	1.94mm	1.97mm	2.02mm
Number and percentage of measured knees with difference <4mm, n (%)	129 (86.0%)	67 (89.3%)	62 (82.7%)
Number and percentage of measured knees with difference <8mm, n (%)	150 (100%)	75 (100%)	75 (100%)

Regression analysis also showed that among the demographic variables collected (sex, age, and laterality), only sex was a significant factor associated with ATJL. Therefore, sex-specific formulas were also derived to be tested for accuracy. Using linear regression analysis, the following regression equations were obtained for each sex:

Females: $ATJL = 0.46(\text{TEW}) + 6.9 \, \text{mm}$

Males: $ATJL = 0.49(\text{TEW}) + 5.9 \, \text{mm}$

where ATJL in millimetres is the dependent variable, TEW in millimetres is the independent variable, and 6.9mm and 5.9mm are the Y-axis intercepts for females and males, respectively.

From the sex-specific formulas, the combined mean difference of the calculated ATJL from the measured ATJL was 2.59 mm with a standard deviation of 1.97 mm. The sex-specific formulas estimated the JL to be within 4 mm of the native JL in 122 (84.7%) measured knees and within 8 mm in 149 (99.3%) measured knees.

Using the fixed distance method, the mean difference between the estimated MEJL and the measured MEJL was 6.57 mm with a standard deviation of 3.23 mm. The JL was estimated to be within 4 mm of the native JL in 45 (30%) measured knees and within 8 mm in 104 (69.3%) measured knees.

## Discussion

In revision TKAs (rTKAs), loss of femoral condyles may be caused by aseptic loosening, infection, erosion by loose components or acrylic cement spacers, periprosthetic fractures, or maybe iatrogenic during implant and/or cement removal. This loss of bone and cartilage complicates the task of determining and restoring the native JL position, thus making JL alteration a common problem in rTKAs. Partington et al. reported JL elevation in 79% of rTKAs with an average elevation of 24 mm [5].

The importance of accurate restoration of the native JL in revision TKA has been demonstrated by several authors. Joint line restoration has been shown to be a significant determinant of post-revision TKA range of motion (ROM) [6], to produce better post-operative functional knee scores [7] and greater improvement in post-operative functional knee scores [8]. On the other hand, JL elevation leads to mid-flexion instability [9] and pseudo-patella baja [10], which in turn could cause reduced ROM, increased knee pain [11], and increased patellofemoral contact stress [12]. Meanwhile, JL depression and the resultant patella alta may lead to patellofemoral instability and subluxation [13].

While there is agreement on the importance of JL restoration, opinions are divided among published studies on the required accuracy of JL restoration. According to Hofmann et al., regardless of the reason for revision TKA, there is a significantly worse functional knee score and range of motion when JL is either elevated or depressed greater than 4mm from native JL position in a revision TKA [14]. These findings were, to a degree, confirmed by a cadaveric study that showed no significant differences in knee kinetics, kinematics, or collateral ligament elongation when the JL is elevated by exactly 4 mm [15]. On the other hand, a prospective study reviewing the effects of JL elevation in rTKA found that Knee Society Scores were significantly worse if the JL was elevated greater than 8 mm from the native JL position [5]. This study, however, did not describe the effects of lesser degrees of JL elevation.

In the absence of accurately scaled contralateral knee imaging, several intraoperative methods are used for JL estimation when distal femoral condyle bone loss is present. A meniscal remnant or scar from the meniscectomy performed during the index TKA was thought to be an identifiable and reliable guide to native JL position. Contrarily, it has been reported that even in TKAs with altered JLs, scar tissue forms at the level of the recreated JL, which may be mistaken for a meniscal scar [16] and therefore misguide JL position during revision TKA.

Fixed distances between the JL and surrounding bony landmarks such as medial and lateral epicondyles [2,17], fibula head [18], tibial tuberosity, and inferior pole of patella have been used to estimate native JL position. The fixed distance used is usually derived from the population mean. Therefore, the further a knee is from mean dimensions, the lower the accuracy and reliability of this method. Medial epicondyle, lateral epicondyle, and tibial tuberosity to JL distances vary significantly between genders [19, 20], while the fibula head apex to JL distance showed wide interindividual variability [21], making them unsuitable for JL estimation. In the current study, we found that the use of a fixed distance of 31 mm as previously reported (2) to estimate MEJL resulted in only 30.0% of estimated MEJL falling within 4 mm of measured MEJL.

Mathematical ratios or formulas aim to exploit the innate correlations between periarticular knee measurements. These correlations remain relatively constant despite variations in knee size and therefore should enable more reliable estimation of the native JL. From the bony landmarks present around the knee, several authors have found the ATJL to TEW correlation to be the strongest [3,4,22]. The use of the ATJL to TEW ratio to estimate JL position was first proposed by Iacono et al. [3] based on measurements taken on plain radiographs. The same author went on to confirm this ratio with intraoperative measurements [23]. It is worth noting that these intraoperative measurements were taken in patients who were undergoing TKA. Such knees presumably would have had significantly reduced and/or abnormal articular cartilage. At approximately the same time, Luyckx et al., employing the same measurements on plain radiographs of healthy knees, also published a similar ratio [4].

These three initial studies and several later studies [24,25], failed to account for the contribution of normal medial femoral condyle cartilage thickness to native JL position, either because of the choice of imaging modality or by intraoperative measurements of diseased knees. Given that the average distal medial femoral condyle cartilage thickness is 2.13 mm [26], we believe that it is imperative for cartilage thickness to be incorporated into any formula for JL estimation in order to mitigate JL elevation. This rings particularly significant when considering the need for precise JL restoration, which is to avoid alteration of >4mm in either direction [14] and that the vast majority of JL alteration in rTKA involves elevation rather than depression [5,27].

This study found that ATJL is significantly influenced by sex. Despite this, the sex-specific formulas that we derived showed only marginal differences in accuracy and reliability when compared to the non-sex-specific formula. This suggests that the larger ATJL seen in male patients is mainly due to larger knees and has less to do with sex-specific differences in the ATJL and TEW correlation. For this reason and in the interest of producing a practical solution to JL restoration, we have chosen to apply one formula for estimation of native JL, irrespective of sex:

$ATJL = 0.53(\text{TEW}) + 2.4 \, \text{mm}$

Formula calculated ATJL was compared to measured ATJL for each MRI, and all differences were analysed according to the critical cut-off values of 4 mm and 8 mm JL alteration as previously quoted by other authors [5,14]. The formula was able to estimate JL within 4 mm of the native JL in 86.0% of measured knees and within 8 mm in 100% of measured knees. In comparison, JL estimation using a fixed MEJL distance produced significantly less accurate results.

The adductor tubercle is a suitable bony landmark for JL restoration as it is easily identifiable on imaging studies as well as intraoperatively and has also demonstrated high intra- and inter-observer reliability [3,28]. The three periarticular bony landmarks necessary for the application of this formula, namely the medial epicondyle, lateral epicondyle, and adductor tubercle, are all preserved in the most common femoral defect types of Anderson Orthopaedic Research Institute (AORI) F2A and F2B [29] and may only be absent in AORI F3 defects.

There are several limitations to this current study. The derived formula was not tested on an independent set of MRI or intraoperative measurements to determine accuracy and precision. It was also noted that male participants showed a slightly larger mean and standard deviation of the difference between calculated and measured ATJL. It may be that the accuracy and precision of the current formula reduce with increasing knee size, but this can only be confirmed or refuted with a larger sample size. It is also beyond the scope of this study to investigate the clinical impact of usage of the current formula when compared to other methods of JL restoration. Further research is necessary to determine if, in fact, the use of this formula yields better outcomes in terms of knee function, pain, and patient satisfaction.

## Conclusions

The formula derived from the current study can be used to reliably estimate native JL position within a clinically significant range in the presence of distal femoral condyle bone loss. Intraoperative estimation of ATJL is possible by measuring TEW and applying the current formula. This formula-derived ATJL can then be used to guide distal femur reconstruction.
